# Supplementary material for: Identification of photoperiod-induced specific miRNAs in the adrenal glands of Sunite sheep (Ovis aries)
Source: Front Vet Sci. 2022 Jul 22;9:888207. doi: 10.3389/fvets.2022.888207 (PMC9354845; doi:10.3389/fvets.2022.888207)
Supplement: Supplementary file 1 [file Data_Sheet_1.ZIP › Supplementary file/miRDeep2 file.zip!/miRDeep2 file/Novel_Structure/LP42A2/Novel_66.pdf]

Provisional ID	: NC_019482.2_36116
Score total	: 23.8
Score for star read(s)	: 3.9
Score for read counts	: 17
Score for mfe	: 1.3
Score for randfold	: 1.6
Score for cons. seed	:
Total read count	: 45
Mature read count	: 44
Loop read count	: 0
Star read count	: 1

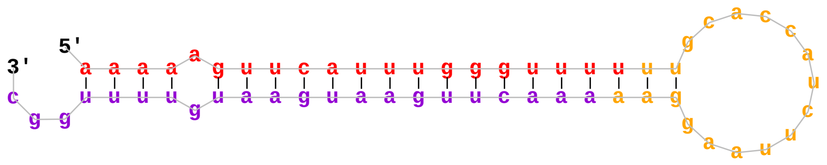

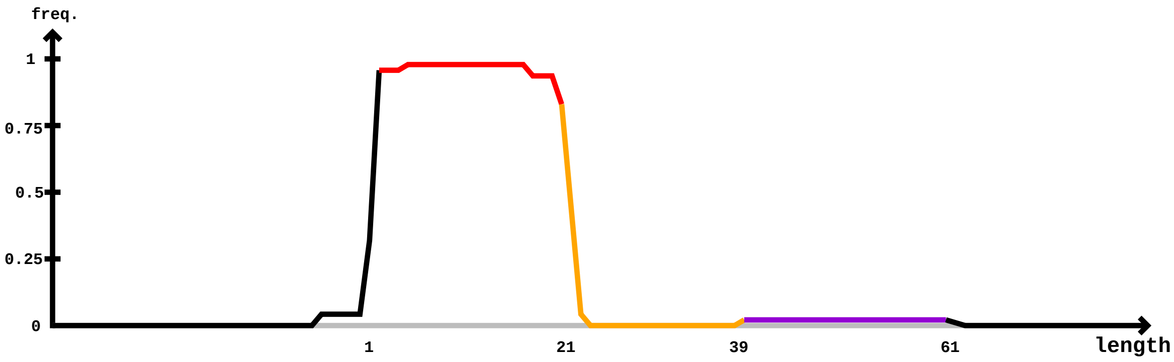


```
                                Mature                             Star
5'- ucccaggcuacccuagguuuugcagguuggccaaaaaguucauuuggguuuuuugcaccaucuuaaggaaaaacuugaaugaauguuuuggccaauacaauauuuuccauuu   -3'    obs
    ucccaggcuacccuagguuuugcagguuggccaaaaaguucauuuggguuuuuugcaccaucuuaaggaaaaacuugaaugaauguuuuggccaauacaauauuuuccauuu          exp
    .((.(((....))).))........(((((((((((.(((((((((((((((((.............))))))))))))))))).)))))))))))................   reads     mm       sample
    ..........................uuggGcaaaaaguucauuuggg................................................................   2         1        seq
    ...............................caaaaaguucGuuuggguuuuu...........................................................   13        1        seq
    ................................aaaaaguucGuuuggguuu.............................................................   5         1        seq
    ................................aaaaaguucGuuuggguuuu............................................................   24        1        seq
    ................................aaaaaguucauuuggguuuuCu..........................................................   1         1        seq
    ...................................aaguucauuCggguuuuuu..........................................................   1         1        seq
    ......................................................................aaacuugaaugaauguuuuggc....................   1         0        seq
```
